# Impact of inhibition of the renin-angiotensin system on early cardiac and renal abnormalities in Sprague Dawley rats fed short-term high fructose plus high salt diet

**DOI:** 10.3389/fnut.2024.1436958

**Published:** 2024-08-22

**Authors:** Sharif Hasan Siddiqui, Rebekah Pitpitan, Boycho Boychev, Dragana Komnenov, Noreen F. Rossi

**Affiliations:** Department of Physiology, Wayne State University, Detroit, MI, United States

**Keywords:** albuminuria, cardiac fibrosis, renal function, fructose, salt, renin angiotensin system

## Abstract

**Introduction:**

The combination of a high fructose and high salt diet typical of western diet induces high blood pressure, aortic stiffening, left ventricular (LV) diastolic dysfunction and impaired renal function in rodents. Despite an activated renin-angiotensin system (RAS) in rats fed high fructose and high salt, acute inhibition of the RAS pathway does not improve cardiac and vascular parameters. It may well be that longer term treatment is required to permit remodeling and improve cardiovascular function. Thus, we hypothesized that chronic RAS inhibition fructose+high salt-fed rats to restore blood pressure (BP) to levels similar to glucose plus normal salt-fed controls will improve cardiorenal function and histopathology.

**Methods:**

Male and female Sprague Dawley rats monitored by hemodynamic telemetry were fed 0.4% NaCl chow during baseline, then changed to chow containing either 20% glucose+0.4% NaCl (G) or 20% fructose+4% NaCl (F) and treated with vehicle, enalapril (Enal, 4 mg/kg/d) or losartan (Los, 8 mg/kg/d) by osmotic minipump for 25–26 days.

**Results:**

BP was elevated in the fructose+high salt groups of both sexes (*P* < 0.05) and restored to control levels by Enal or Los. Pulse wave velocity (PWV) was lower in female F+Los rats and cardiac output higher in female F+Enal rats. GFR was not changed by diet or treatment. Fructose+high salt groups of both sexes displayed higher albuminuria that was decreased by Enal in male rats. Cardiac fibrosis and mesangial hypercellularity were greater in fructose+high salt-fed rats of both sexes and improved with either Los or Enal.

**Discussion:**

Thus, inhibition of the RAS improves early changes in cardiac and renal histopathology in both sexes and albuminuria in male rats fed high fructose and high salt diet. Functional improvements in cardiorenal parameters may require longer treatment.

## 1 Introduction

The presence of cardiac and renal dysfunction is a worldwide phenomenon observed in the United State, Europe, Asia and Africa (1–3). The close interaction between cardiac and renal function has been recognized for more than 200 years (4, 5). The heart and kidneys play crucial roles in the regulation of blood volume, blood pressure and tissue perfusion. For example, the prevalence of left ventricular hypertrophy and diastolic dysfunction is increased in individuals with chronic kidney disease as is the risk of a major adverse cardiovascular event or mortality (6–8). Conversely, up to one-third of individuals with heart failure develop worsening renal function (9, 10) with the greatest risk observed in individuals with heart failure and preserved ejection fraction (11). Often it is unclear which organ was affected first in cardiorenal disease patients (2), and it is altogether possible that cardiac and renal damage occur concurrently due to a common etiology (12).

Traditional risk factors such as diabetes, obesity, smoking and hypertension account for only approximately 40% of individuals with cardiac and renal disease (13, 14) particularly in individuals with reduced access to nutritious food due to socioeconomic stressors (15). Neurohormonal dysregulation, abnormal metabolism and oxidative stress may all contribute to worsening cardiac and renal function (14, 16). Studies suggest that the increase in consumption of fructose, spurred by the use of high-fructose corn syrup in beverages and processed foods, together with high salt intake strongly contributes to the development of cardiometabolic and renal disease (17, 18). Preclinical data in rats indicate that the concurrent ingestion of fructose limits urinary sodium excretion and raises blood pressure. Thus, fructose intake can induce salt sensitivity of blood pressure (19–21). Notably, salt sensitivity of blood pressure is associated with increased cardiovascular mortality even in normotensive individuals (22).

Arterial stiffness (23–25) and insulin resistance (26, 27) are recognized as independent risk factors for the development of cardiovascular and renal disease in humans. Male rats ingesting 20% fructose (w/v) in the drinking water and 4% NaCl in the rat chow for 3 weeks display normal serum glucose levels but exhibit insulin resistance, elevated blood pressure, reduced aortic compliance and elevated pulse wave velocity (PWV), whereas blood pressure, aortic compliance and left ventricular function are not altered in fructose and high salt-fed female rats (28–30). In a murine model, ingestion of much higher fructose (60% w/v) for 12 weeks induces left ventricular hypertrophy and cardiac fibrosis (31) thereby impairing cardiac function (32). Limited data are available on renal histopathology, but renal mesangial cell hyperplasia has been demonstrated with a fructose-rich diet (33). In Dahl salt-sensitive rats, fructose (60% w/v) diet for 12 weeks results in glomerular sclerosis, afferent arteriolar thickening and interstitial fibrosis (34). Glomerular filtration rate (GFR) is significantly lower and urine albumin excretion is higher in male rats ingesting rat chow with 20% caloric intake as fructose plus 4% NaCl for 12 weeks compared with rats on a 20% glucose with 0.4% NaCl diet.

Fructose-fed male rats exhibit higher plasma renin activity, hepatic angiotensinogen and plasma angiotensin II levels (Ang II) (35). Expression of both angiotensin converting enzyme (ACE) and angiotensin type 1 receptor (AT_1_R) mRNA is upregulated in cardiac and aortic tissues with high fructose feeding in male but not female rats (36). Concurrent intake of high fructose and high salt diet augments renal sympathetic nerve activity (28) and prevents the suppression of renin despite higher arterial pressure and expanded extracellular volume (20). Renal denervation normalizes blood pressure, decreases renin and Ang II, and improves insulin resistance (28). Acute administration of the central sympathetic inhibitor clonidine improves aortic stiffness in the male rats. Despite a decline in arterial pressure comparable to that observed with clonidine, acute blockade of the renin-angiotensin system (RAS) with either losartan or enalapril does not improve PWV (30). It is not known if inhibition of the RAS system requires a more prolonged treatment to prevent cardiac, vascular and/or renal histopathology and function. Moreover, there are scant data on the functional impact of fructose and high salt diet on cardiorenal parameters in female rats.

Thus, we hypothesized that interruption of RAS activity for 3 weeks by either inhibition of ACE or blockade of AT_1_R in male rats fed a high fructose, high salt diet such that MAP will be brought to a level identical to that of control glucose plus normal salt-fed Sprague Dawley rats will improve histopathological indices of cardiac fibrosis and mesangial hypercellularity as well as ameliorate left ventricular diastolic function, PWV, insulin sensitivity, GFR and albuminuria. We further hypothesize the high fructose plus high salt diet will result in little or no deleterious effects on cardiac, vascular or renal parameters in female rats.

## 2 Experimental design and methods

### 2.1 Animals

All rats were cared for in compliance with the principles of the National Research Council Committee *Guide for the Care and Use of Laboratory Animals*. The Wayne State University Institutional Animal Care and Use Committee approved the protocol and all amendments of the animal research plan. Experiments were performed on male and female Sprague Dawley rats (Envigo, Indianapolis, IN) that were 12 ± 1 week-old. Rats were permitted to acclimate for 48 h under controlled housing conditions (21–23°C; 06:00–18:00 light cycle; 18:00:06:00 dark cycle). They were permitted *ad libitum* access to standard rat chow containing 0.49% NaCl and water (Envigo Teklad, Indianapolis, IN) until initiated into the experimental protocol. Since our previous work demonstrated similar PWV and left ventricular diastolic function in glucose plus high salt or fructose plus normal salt diets compared with control glucose plus normal salt, we compared glucose normal salt rats (control group) with fructose high salt rats either with or without pharmacological treatment in accordance with the principle of reducing the number of animals used.

### 2.2 Protocols

**Figure 1:**
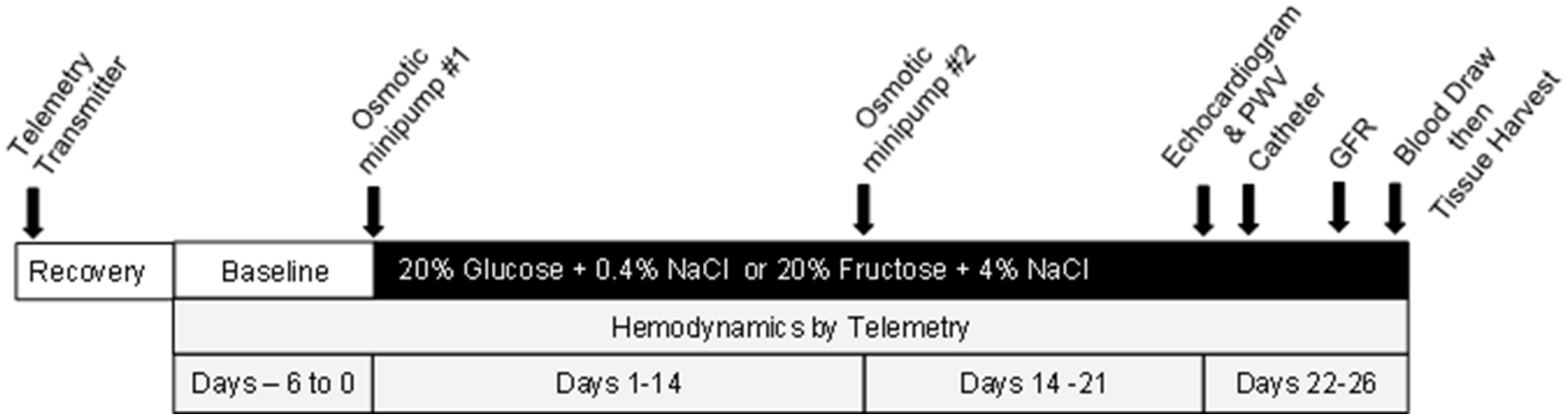
Schematic of methodology of this study.

The overall protocol is illustrated in Figure 1 (also see Supplementary Figure S1). After the two-day acclimation, rats underwent surgical placement of hemodynamic radiotransmitters (see below for all surgical procedure details) and permitted to recover, then baseline arterial pressures and heart rate were recorded for 6 days during which all rats were on *ad libitum* water plus 20% glucose plus 0.4% NaCl chow (TestDiet #1817915-209, Richmond, IN). Rats were then anesthetized and an osmotic minipump (Alzet 2002, Cupertino, CA) was inserted subcutaneously under isoflurane anesthesia to deliver either vehicle or the drug at a dose to bring MAP to a level as close to identical to that of the glucose-fed control group (37, 38). Due to different vehicle treatments required, two separate sets of rats were studied. Set one included the following: 20% glucose and 0.4% NaCl chow plus minipump to deliver isotonic saline vehicle (G + V), 20% fructose and 4% NaCl plus isotonic saline vehicle (F + V); and 20% fructose and 4% NaCl plus minipump delivering losartan 8 mg/kg/day (F + Los). Set two included 20% glucose and 0.4% NaCl chow plus isotonic saline: dimethylsulfoxide (DMSO) at a 1:1 proportion (G + V + D), 20% fructose and 4% NaCl plus saline: DMSO (F + V + D), and 20% fructose and 4% NaCl diet plus minipump to deliver enalapril 4 mg/kg/day (F + Enal) (TestDiet #1818296-209, Richmond, IN). The nutritional composition of the experimental diets are presented in Supplementary Tables S1, S2. Based on our previous study showing improvement in cardiovascular parameters with clonidine, we initially incorporated a third set of rats with clonidine 0.4 mg/kg/day (39) in the design but needed to eliminate this group due to complications resulting from diminished bowel motility (40). Vehicle or drug was administered over the course of (25–26 days). All chemicals and drugs were obtained from Millipore Sigma (Milwaukee, WI). Injection solutions were sterilized by passage through a Millex 0.22 mm filter (Millipore Sigma).

On day 21, each rat underwent echocardiography and evaluation of PWV under 2.5% isoflurane anesthesia. The next day, a vascular catheter was placed into the left carotid artery under ketamine 80 mg/kg and xylazine 10 mg/kg i.p. anesthesia for subsequent blood draws. Three days later, glomerular filtration rate (GFR) was assessed transdermally using FITC-sinistrin (MediBeacon, St. Louis, MO). One or two days later, blood was obtained from conscious rats for measurement of glucose and insulin. Immediately thereafter, they were anesthetized with sodium pentobarbital 40 mg/kg i.p. Urine was obtained directly from the bladder for subsequent assessment of albumin, and heart, aorta, and kidney were harvested and fixed in 10% formalin for histologic evaluation.

### 2.3 Surgical procedures

All rats received preemptive analgesia with Ethiqa 0.65 mg/kg s.c. (Fidelis, New Brunswick, NJ) prior to each surgical procedure.

#### 2.3.1 Telemetry transmitter placement

Rats were anesthetized with ketamine/xyalazine as noted above. Briefly, the right inguinal area was prepped, the femoral artery was isolated and the gel-filled catheter of the hemodynamic transmitter (HD-S10, Data Sci. Intl., New Brighton, MN) was inserted and then advanced into the iliac and terminal aorta. The catheter was secured with 3-0 silk sutures (Ethicon, Johnson & Johnson, New Brunswick, NJ). The body of the transmitter was tunneled subcutaneously and stabilized with tissue adhesive (Vetbond, 3 M, Neuss, Germany). The incision was closed with 3-0 nylon sutures (Ethicon).

#### 2.3.2 Osmotic minipump placement

Rats were anesthetized with 3% isoflurane in an induction chamber followed by 2% isoflurane by nose cone. They were placed in prone position on a heated platform. An osmotic minipump (Alzet 2002, Cupertino, CA) was inserted subcutaneously via a 1 cm incision in the dorsal area just to the left of the vertebrae and the incision was sutured. This osmotic pump was designed to deliver 0.5 mL/h for 14 days and the pump was primed to initiate infusion prior to insertion by incubating in saline at 37°C for 4 h prior to placement. On day 14, a second, identical osmotic minipump was inserted following the same procedures but placed to the right of the vertebral column. We changed to the two-pump protocol for all animals reported herein upon the recommendation of our veterinarian.

#### 2.3.3 Vascular catheter placement

After ketamine/xylazine anesthesia, the left carotid artery was exposed, and the catheter was inserted, secured with 3-0 silk suture then tunneled subcutaneously and exteriorized at the base of the neck posteriorly as previously described (41).

#### 2.3.4 Echocardiography

The same investigator (DK), blinded to the group assignment of the animals, performed all echocardiograms and PWV (Vevo 3100, Visual Sonics, Bothell, WA) by methods previous reported from Komnenov et al. (30). Rats were anesthetized with 3% in the induction chamber then maintained at 1.5% isoflurane by inhalation via nose cone. Rats were supine on a heated platform with all four limbs secured to the ECG electrodes by adhesive tape. Fur overlying the thorax was shaved and followed by applying a depilatory cream for 30 s (Nair, Pharmaceutical Innovations). Body temperature was monitored by rectal probe and maintained at 37°C. Echocardiographic recordings were performed using standard techniques. Images were acquired via the MX-250S transducer to obtain a parasternal short axis view in M-mode at the level of the papillary muscles to ascertain systolic function and left ventricular dimensions. Left ventricular diastolic function was assessed using pulse wave Doppler recordings of transmitral flow velocities aligned in the apical four chamber view. Aortic PWV was determined using B-mod and pulse wave Doppler of aortic flow velocities at two positions along the aortic arch. The distance between the two positions was determined from the B-mode image. The distance was then divided by the transit time from position one to position two calculated using the ECG tracing as a reference.

#### 2.3.5 Glomerular filtration rate

GFR was measured using bolus injection of fluorescein-isothiocyanate (FITC)-sinistrin as described by Schock-Kusch et al. (42) and previously used by our laboratory (19). Rats were briefly anesthetized with 3% isoflurane followed by 1.5% isoflurane. A small area of skin ~1.5 cm diameter was shaved and remaining fur was removed with a depilatory cream as described above. The LED-emitting optical transducer attached to an adhesive patch was positioned on the skin. Baseline measurements were recorded for about 3 min. Then, FITC-sinistrin, 5 mg/100 mg BW (Fresenius-Kabi, Lake Zurich, IL) was injected via catheter or tail vein and the animal was permitted to awaken. Recordings were taken for 2 h. FITC-sinistrin disappearance kinetics were analyzed over the first 90 min after injection via a three-compartment model. GFR was calculated using MB Studio software (MediBeacon, St. Louis, MO) as previously described (19, 42).

### 2.4 Terminal procedures

Eighteen to twenty-four hours after completion of GFR measurements, blood samples were obtained in conscious rats between 09:00–10:00. Glucose levels were measured on ~50 mL whole blood using a One-Touch Ultra glucose monitor (LifeScan, Inc., Malvern, PA) collected directly from the arterial catheter. For insulin assay, 1 mL blood was collected directly from the catheter into prechilled tubes containing 50 mL ethylenediamine tetra-acetic acid, immediately centrifuged at 3,000 rpm for 10 min at 4°C. Plasma was stored at −70°C until assay.

Rats were euthanized with sodium pentobarbital 120 mg/kg i.v. Urine was obtained directly by aspirating from the bladder. Hearts and kidneys were harvested, maintained in ice-cold saline, and weighed. Urine as well as samples of heart and kidney tissue were frozen and stored at −70°C. The remainder of heart and kidney tissue was placed into 10% formalin for 48 h and processed for paraffin embedding.

### 2.5 Histologic analyses

Ten micrometer sections of kidney were stained with periodic acid Schiff (PAS) according to the manufacturer’s directions (Newcomer Supply, Middleton, WI). Morphometric analysis of glomerular mesangial cellularity was performed in accordance with the Oxford classification (43). Forty glomeruli were analyzed per rat. Briefly, mesangial cellularity was scored as follows: normal <4 mesangial cells/mesangial area, mild 4–5 mesangial cells/mesangial area, moderate 6–7 mesangial cells/mesangial area, and severe ≥8 mesangial cells/mesangial area. The score for a given glomerulus was taken as that of the most cellular area for that glomerulus. The region at the vascular pole was not included in the analyses.

Cardiac tissue was stained with picrosirius red per manufacturer’s recommendations (Polysciences, Inc., Warrington, PA). Cardiac fibrosis was determined using ImageJ as the percentage of the area of picrosirius red staining vs. total tissue area of the left ventricle. All morphometric analyses were performed by a researcher blinded to the group assignment.

### 2.6 Hormonal and chemical assays

Plasma insulin was determined using rat insulin ELISA kit (Life Technologies-Invitrogen, Waltham, MA). Urine albumin was measured by albumin ELISA assay (Abnova, Walnut, CA). Creatinine was evaluated via standard colorimetric assay (Cayman, Ann Arbor, MI).

### 2.7 Statistical analyses

All data are presented as mean ± SE. The number of animals per group in the study (*n* = 6) was determined to achieve a power of 90% at an α-level of 0.05 to identify a difference of 100 mm/s in PWV with a standard deviation of 40 mm/s based on values from previous studies with this model (19, 30). Comparisons between baseline and post-treatment body weight in the same animal were performed by paired *t-*test. Comparisons among groups was accomplished by one-way ANOVA for multiple comparisons using Holm-Sidak *post hoc* analysis. Not all animals had sufficient urine in the bladder for collection. When *n* values deviate from the original assignment, the reason for missingness is provided and values imputed as the mean. A *p*-value <0.05 was considered significant.

Except for comparisons of body weight and organ weights at the beginning and the end of the study which were assessed by two-way ANOVA, the present study was not designed or powered to evaluate differences between sexes, but rather to assess the impact of RAS inhibition on fructose plus high salt on cardiovascular and renal parameters within a given sex.

## 3 Results

### 3.1 Metabolic and humoral parameters

Initial body weights of rats of the same age were significantly lower in female vs. male rats in each of the groups (*p* < 0.01) (Table 1). The difference between male and female rat weights was maintained throughout the study such that final body weights also differed significantly between male and female rats receiving the same treatment (*p* < 0.01). The final body weights of both male and female rats differed significantly from the initial body weight on each of the dietary regimens, but there was no difference between the weights of control glucose plus normal salt vs. fructose plus high salt-fed rats of either sex. Kidney and heart weights were similar among rats of the same sex and protocol, but kidney weights were significantly lower in each of the female vs. male rats within the same treatment group (*p* < 0.01). Non-fasting glucose did not differ among the groups. Plasma insulin was significantly lower in the male F + V group compared with the male G + V group. As well as the insulin was significantly higher in the male F + Enal (29.8 ± 5.0 mIU/mL) compared with F + V + D male rats (14.2 ± 1.6 mIU/mL) (*p* < 0.05). The insulin levels of female rats did not differ among the dietary groups. The plasma glucose:insulin ratio was significantly higher in the F + V + D group compared with the G + V + D group in male rats, but not differences were observed in the female rats (Table 1).

**Table 1:** Baseline and final body weights, heart and kidney weights, blood glucose, insulin and glucose:insulin ratio in glucose + normal salt and fructose + high salt fed rats in losartan and enalapril treated groups of both sexes.

	*n*	Initial body weight (grams)	Final body weight (grams)	Both kidneys weight (grams)	Heart weight (grams)	Non-fasting glucose (mg/dL)	Insulin (μIU/mL)	Glucose: insulin ratio (nM/nM × 10^6^)
Males								
G + V	6	264.3 ± 9.1	308.3 ± 14.2^†^	1.953 ± 0.056	1.203 ± 0.127	165 ± 21	25.9 ± 2.9	60.9 ± 9.7
F + V	6	256.8 ± 4.4	302.2 ± 11.8^†^	2.013 ± 0.062	1.110 ± 0.057	136 ± 6	19.0 ± 0.9^*^	69.2 ± 8.1
F + Los	6	244.2 ± 7.9	302.0 ± 8.9^†^	2.232 ± 0.208	1.091 ± 0.030	140 ± 13	19.9 ± 2.5	55.4 ± 12.5
G + V + D	6	252.2 ± 10.6	319.5 ± 15.0^†^	1.960 ± 0.159	1.171 ± 0.048	132 ± 13	23.5 ± 1.4	49.5 ± 3.0
F + V + D	6	248.7 ± 8.9	305.8 ± 8.5^†^	2.118 ± 0.041	1.152 ± 0.029	124 ± 12	14.2 ± 1.6	72.5 ± 5.6^*^
F + Enal	6	254.5 ± 7.0	303.2 ± 5.3^†^	2.084 ± 0.164	1.102 ± 0.084	147 ± 12	29.8 ± 5.0	57.2 ± 4.5
Females								
G + V	6	234.3 ± 3.9	256.0 ± 5.2^†#^	1.571 ± 0.059^#^	0.991 ± 0.041	138 ± 8	14.8 ± 2.5	85.1 ± 14.1
F + V	6	233.2 ± 6.4	250.2 ± 5.9^†#^	1.689 ± 0.090^#^	0.954 ± 0.039	129 ± 7	20.6 ± 1.5	68.9 ± 3.0
F + Los	6	230.0 ± 5.1	254.8 ± 6.1^†#^	1.594 ± 0.070^#^	0.953 ± 0.046	156 ± 13	16.9 ± 0.6	86.6 ± 9.6
G + V + D	6	240.0 ± 3.1	249.7 ± 5.6^†#^	1.560 ± 0.059^#^	1.001 ± 0.030	142 ± 14	16.4 ± 1.9	81.6 ± 6.0
F + V + D	6	227.2 ± 2.0	241.8 ± 6.6^†#^	1.564 ± 0.073^#^	1.009 ± 0.032	149 ± 16	23.7 ± 4.4	68.2 ± 12.6
F + Enal	6	230.0 ± 4.1	251.8 ± 3.8^†#^	1.655 ± 0.077^#^	0.930 ± 0.038	159 ± 11	18.8 ± 3.3	88.2 ± 13.7

G + V, 20% glucose + 0.4% NaCl, saline vehicle treated; F + V, 20% fructose + 4% NaCl, saline vehicle; F + Los, 20% fructose + 4% NaCl, losartan 8 mg/kg/day; G + V + D, 20% glucose + 0.4% NaCl, saline:DMSO 1:1 vehicle; F + V + D, 20% fructose + 4% NaCl, saline:DMSO 1:1 vehicle; F + Enal, 20% fructose + 4% NaCl, enalapril 4 mg/kg/day. Values are mean ± SE. ^*^p < 0.05 vs. G + V same sex; ^†^*p* < 0.05 vs. baseline. ^§^*p* < 0.05 vs. F + V + D same sex; ^#^*p* < 0.01 vs. same dietary group, male rats.

### 3.2 Systemic blood pressure and heart rate

**Figure 2:**
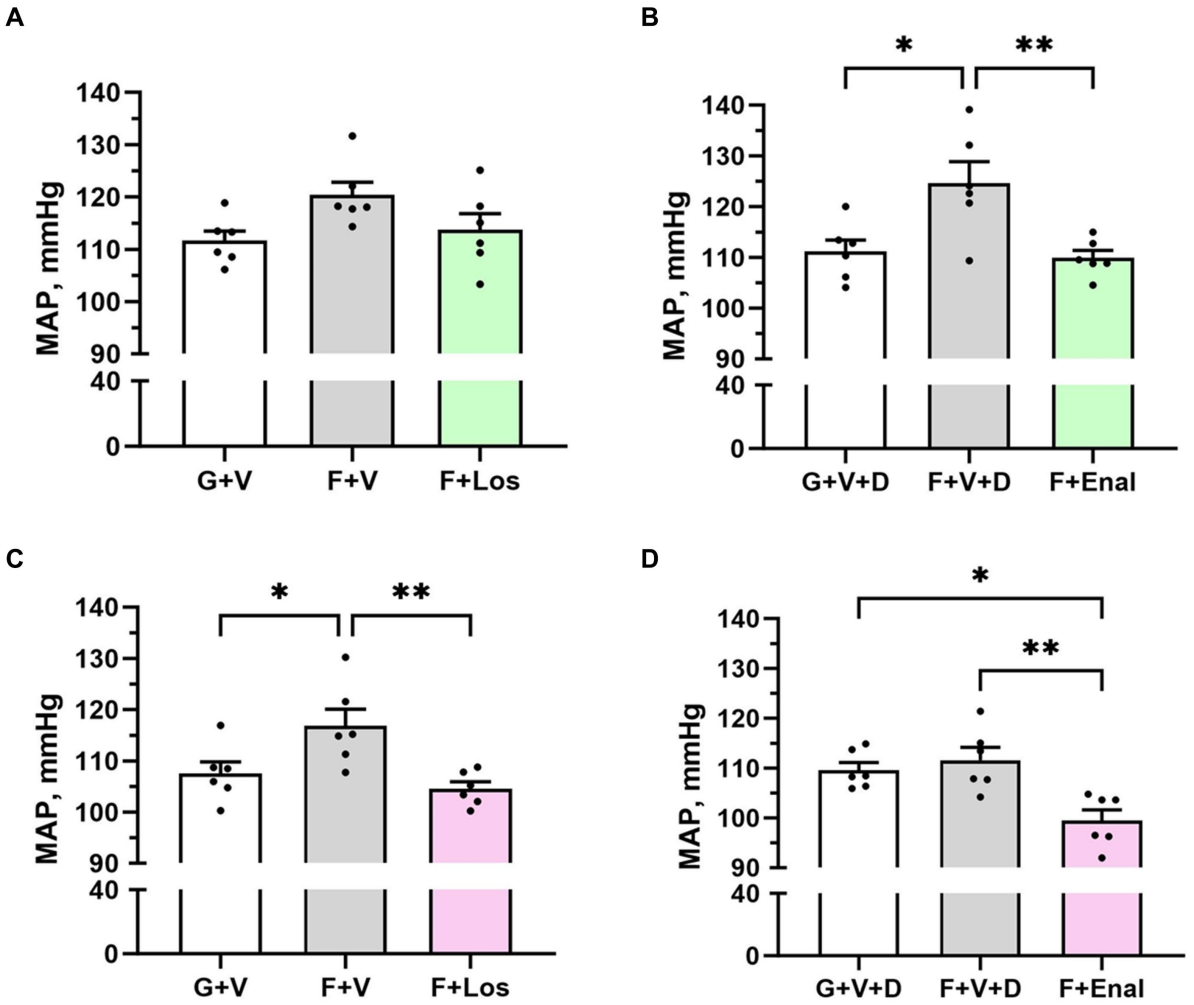
Mean arterial pressure (MAP) in male **(A,B)** and female **(C,D)** rats fed for 3 weeks on 20% glucose plus 0.4% NaCl (G) or 20% fructose plus 4% NaCl chow (F) and concurrently treated with either saline vehicle (V) or losartan (Los, 8 mg/kg/day s.c.): G + V, F + V, and F + Los; or saline:DMSO vehicle (V + D) or enalapril (Enal, 4 mg/kg/day s.c.): G + V + D, F + V + D, and F + Enal by osmotic minipump. Values are mean ± SE; *n* = 6 for each group. ^*^p < 0.05 and ^**^p < 0.01 by ANOVA.

Vehicle-treated fructose plus high salt-fed male rats displayed significantly higher systolic blood pressures than their respective glucose plus normal salt control group (Table 2). Systolic blood pressure in the female F + V group was significantly greater than its G + V control, but the female F + V + D rats did not exhibit higher systolic pressure compared with the female G + V + D group. Figure 2 shows that the same pattern emerged for MAP in both male and female rats. MAP in the male F + V group was higher than the G + V group but did not achieve significance (*p* < 0.065). Diastolic blood pressure was elevated only in male F + V + D rats. As per experimental design, treatment with either losartan or enalapril restored systolic and MAP to levels no different from G + V or G + V + D groups in both male and female rats with the exception of female rats treated with enalapril (F + Enal) where both systolic and MAP were significantly lower than either G + V + D or F + V + D groups (Figure 2). Diastolic blood pressure was significantly lower in male F + Enal rats and female F + Los rats compared with their respective fructose-fed groups. Diastolic blood pressure in the female F + Enal group was lower than either female G + V + D or F + D + V rats. Heart rates did not differ among either male or female groups except for lower heart rate in F + V vs. G + V female rats (Table 2).

**Table 2:** Baseline and post-treatment systolic and diastolic blood pressures and heart rates glucose + normal-salt and fructose + high salt fed rats rats in losartan and enalapril treated groups of both sexes.

	*n*	Baseline SBP (mmHg)	Post-treatment SBP (mmHg)	Baseline DBP (mmHg)	Post-treatment DBP (mmHg)	Baseline HR (bpm)	Post-treatment HR (bpm)
Males							
G + V	6	129.5 ± 2.3	124.4 ± 2.4	92.8 ± 1.8	94.4 ± 3.1	381 ± 8	371 ± 5
F + V	6	134.0 ± 3.2	138.9 ± 2.7^*^	91.0 ± 1.5	99.2 ± 2.3	393 ± 10	367 ± 3
F + Los	6	131.7 ± 1.8	125.1 ± 2.8^§^	89.9 ± 1.8	93.7 ± 3.0	400 ± 10	371 ± 5
G + V + D	6	131.2 ± 12.4	134.0 ± 3.2	90.5 ± 1.7	92.0 ± 1.8	394 ± 8	375 ± 4
F + V + D	6	129.7 ± 2.6	146.4 ± 4.9^*†^	93.4 ± 2.2	104.0 ± 3.7^*†^	387 ± 6	370 ± 3
F + Enal	6	136.4 ± 1.9	133.6 ± 1.6^§^	92.1 ± 1.2	89.8 ± 1.1^§^	383 ± 8	375 ± 8
Females							
G + V	6	128.1 ± 2.4	127.7 ± 3.8	88.7 ± 1.5	90.4 ± 2.4	406 ± 5	384 ± 10
F + V	6	130.4 ± 2.1	142.7 ± 5.4^*†^	89.7 ± 1.5	97.4 ± 2.9	390 ± 8	344 ± 7^†^
F + Los	6	134.8 ± 1.8	128.7 ± 1.8^§^	91.0 ± 2.0	84.1 ± 1.3^§^	411 ± 8	395 ± 5
G + V + D	6	129.5 ± 1.6	132.6 ± 2.4	89.3 ± 1.4	89.8 ± 1.6	390 ± 9	382 ± 5
F + V + D	6	129.9 ± 2.3	134.7 ± 2.9	90.7 ± 1.7	91.5 ± 2.2	386 ± 8	373 ± 2
F + Enal	6	133.3 ± 1.5	121.6 ± 2.6^*§^	91.9 ± 1.9	80.8 ± 2.2^*§^	396 ± 6	384 ± 4

SBP, systolic blood pressure; DBP, diastolic blood pressure; HR, heart rate. Other abbreviations as in Table 1. Values a mean ± SE. ^*^p < 0.05 vs. glucose-fed same group and sex; ^†^*p* < 0.05 vs. baseline; *p* < 0.05 vs. fructose-fed vehicle-treated, same group and sex; ^§^*p* < 0.05 vs. F + V + D and F + V same group and sex.

### 3.3 Cardiac echocardiography and histopathology

**Figure 3:**
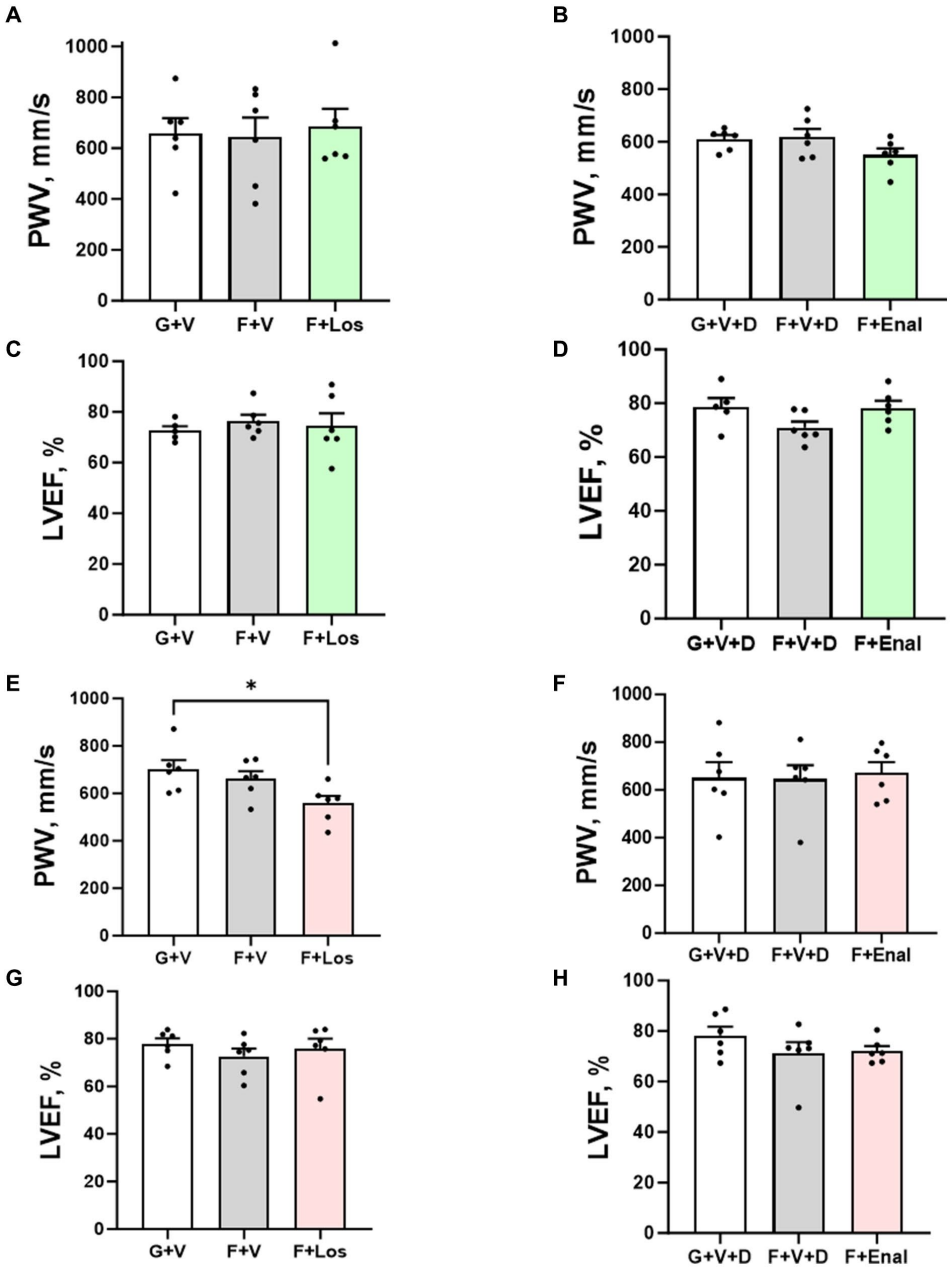
Aortic pulse wave velocity (PWV) and left ventricular ejection fraction (LVEF) in male **(A–D)** and female **(E–H)** rats fed for 3 weeks on 20% glucose plus 0.4% NaCl (G) or 20% fructose plus 4% NaCl chow (F) and concurrently treated with either saline vehicle (V) or losartan (Los, 8 mg/kg/day s.c.): G + V, F + V, and F + Los; or saline:DMSO vehicle (V + D) or enalapril (Enal, 4 mg/kg/day s.c.): G + V + D, F + V + D, and F + Enal by osmotic minipump. Values are mean ± SE; *n* = 6 for each group. ^*^p < 0.05 by ANOVA.

Table 3 depicts the results of echocardiography of LV at the end of the experiment. LV parameters are not significantly different among the groups in either male or female rats except for the cardiac output being significantly higher in the F + Enal group compared with the F + V + D (44.99 ± 3.63 mL/min) group in female rats. LV ejection fraction (Table 3); however, LV ejection fraction was similar across all treatments and in both sexes (Figures 3C,D,G,H). Losartan significantly decreased aortic PWV compared with G + V female rats (Figure 3E). Otherwise, PWV was not altered by 3 weeks of fructose plus high salt feeding across all groups. Losartan and enalapril exerted no effect on PWV in any of the other three groups (Figures 3A,B,F).

**Table 3:** Echocardiographic parameters at the end of treatments in male and female glucose + normal salt and fructose + high salt-fed rats in losartan and enalapril treated groups of both sexes.

Groups						
	G + V	F + V	F + Los	G + V + D	F + V + D	F + Enal
Males						
E/A	1.21 ± 0.12	1.15 ± 0.17	1.33 ± 0.06	1.18 ± 0.05	1.16 ± 0.06	1.11 ± 0.03
HR (BPM)	353 ± 13.5	361 ± 12.05	343 ± 10.17	342 ± 8.38	326 ± 9.99	357 ± 13.77
LVID_S_ (mm)	3.86 ± 0.2	3.21 ± 0.2	3.47 ± 0.39	3.44 ± 0.32	3.79 ± 0.2	3.24 ± 0.2
LVID_D_ (mm)	6.74 ± 0.3	5.95 ± 0.14	6.34 ± 0.24	6.70 ± 0.19	6.46 ± 0.17	6.29 ± 0.24
Fraction shortening (%)	42.9 ± 1.5	46.2 ± 2.54	45.7 ± 4.85	48.96 ± 3.47	41.46 ± 1.93	48.35 ± 2.76
Cardiac output (mL/min)	60.75 ± 5	48.9 ± 2.05	51.91 ± 4.07	61.95 ± 2.82	49.35 ± 2.41	57.17 ± 6.55
LV mass (mg)	632.6 ± 58	641.9 ± 28.6	632.2 ± 29.4	679.4 ± 35.2	639.5 ± 47.0	622.7 ± 70
LVAW_S_ (mm)	2.72 ± 0.1	3.06 ± 0.1	2.90 ± 0.24	3.14 ± 0.1	2.97 ± 0.12	3.05 ± 0.1
LVAW_D_ (mm)	1.77 ± 0.1	1.93 ± 0.1	1.98 ± 0.13	1.87 ± 0.12	1.88 ± 0.12	1.91 ± 0.08
LVPW_S_ (mm)	2.66 ± 0.08	3.01 ± 0.17	2.67 ± 0.1	2.83 ± 0.2	2.57 ± 0.18	2.67 ± 0.31
LVPW_D_ (mm)	1.64 ± 0.07	2.01 ± 0.12	1.69 ± 0.06	1.76 ± 0.09	1.73 ± 0.1	1.68 ± 0.12
Females						
E/A	1.26 ± 0.07	1.16 ± 0.08	1.40 ± 0.08	1.23 ± 0.1	1.27 ± 0.05	1.15 ± 0.09
HR (BPM)	347 ± 10.4	331 ± 9.63	334 ± 10.85	348 ± 9.48	337 ± 5.27	349 ± 5.82
LVID_S_ (BPM)	3.28 ± 0.24	3.76 ± 0.31	3.27 ± 0.4	3.15 ± 0.26	3.60 ± 0.39	3.91 ± 0.22
LVID_D_	6.28 ± 0.3	6.58 ± 0.25	5.98 ± 0.41	6.10 ± 0.19	6.13 ± 0.3	6.79 ± 0.22
Fraction shortening (%)	47.89 ± 2.1	43.24 ± 2.83	46.02 ± 3.57	48.54 ± 3.51	41.87 ± 3.52	42.56 ± 1.75
Cardiac output (mL/min)	54.38 ± 4.5	53.13 ± 2.33	45.3 ± 5.46	50.75 ± 2.83	44.99 ± 3.63	60.09 ± 2.93^*^
LV mass (mg)	514.3 ± 35	598.7 ± 38.1	553.2 ± 61.3	519.5 ± 29.5	555.5 ± 38.2	566.9 ± 45.1
LVAW_S_ (mm)	2.80 ± 0.08	2.64 ± 0.09	2.69 ± 0.01	2.88 ± 0.07	2.69 ± 0.08	2.56 ± 0.1
LVAW_D_ (mm)	1.70 ± 0.07	1.68 ± 0.04	1.76 ± 0.07	1.77 ± 0.05	1.71 ± 0.05	1.63 ± 0.04
LVPW_S_ (mm)	2.57 ± 0.13	2.71 ± 0.09	2.70 ± 0.12	2.65 ± 0.1	2.58 ± 0.09	2.62 ± 0.13
LVPW_D_ (mm)	1.51 ± 0.18	1.71 ± 0.12	1.78 ± 0.2	1.56 ± 0.08	1.76 ± 0.1	1.51 ± 0.08

E/A, ratio of early to late phase ventricular filling; HR, heart rate; CO, cardiac output; LVID_S_, left ventricular systolic internal diameter; LVID_D_, left ventricular internal diastolic internal diameter; LV mass, left ventricular mass; LVAW_S_, left ventricular systolic anterior wall width; LVAW_D_, left ventricular diastolic anterior wall width; LVPW_S_, left ventricular systolic posterior wall width; LVPW_D_, left ventricular diastolic posterior wall width. Values are mean ± SE; *n* = 6 for each group. *p* < 0.05 vs. F + V + D.

**Figure 4:**
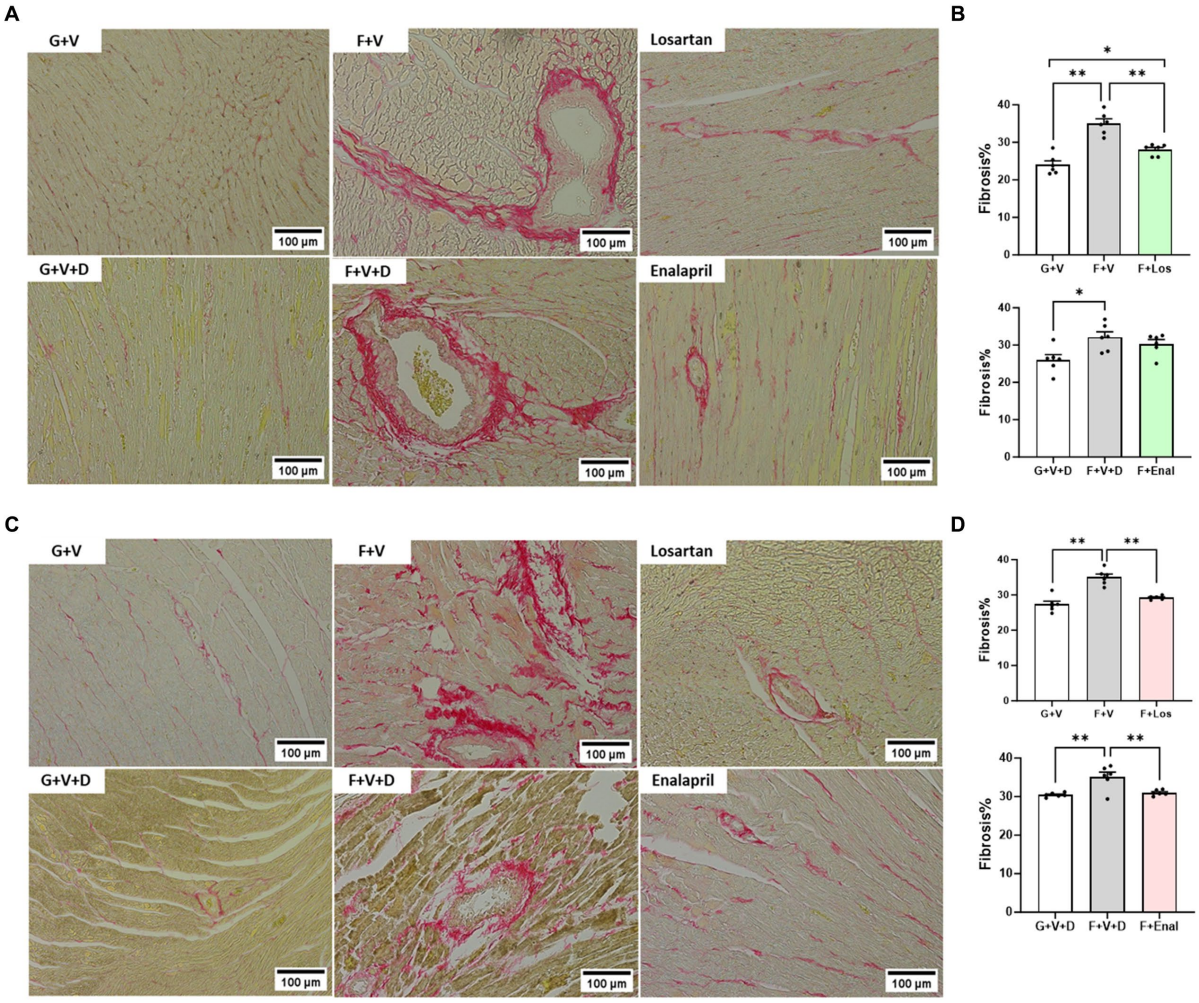
Picrosirius red staining for collagen in cardiac tissue in male **(A)** and female **(C)** rats fed for 3 weeks on 20% glucose plus 0.4% NaCl (G) or 20% fructose plus 4% NaCl chow (F) and concurrently treated with either saline vehicle (V) or losartan (Los, 8 mg/kg/day s.c.): G + V, F + V, and F + Los; or saline:DMSO vehicle (V + D) or enalapril (Enal, 4 mg/kg/day s.c.): G + V + D, F + V + D, and F + Enal by osmotic minipump. Quantitation of fibrosis as percentage of total LV area is shown for males **(B)** and females **(D)**. Values are mean ± SE; *n* = 6 for each group. ^*^p < 0.05 and ^**^p < 0.01 by ANOVA.

Picrosirius red staining as an index of collagen in LV tissue is shown in Figure 3. Fructose plus high salt diet markedly increased interstitial and perivascular fibrosis in vehicle-treated rats of both sexes and was significantly attenuated by AT_1_R blockade (Figures 4A–D). ACE inhibition with enalapril significantly reduced LV collagen deposition in fructose-high salt-fed female rats (Figures 4C,D) but not male rats (Figures 4A,B).

### 3.4 Glomerular filtration rate and glomerular mesangial morphometry

**Figure 5:**
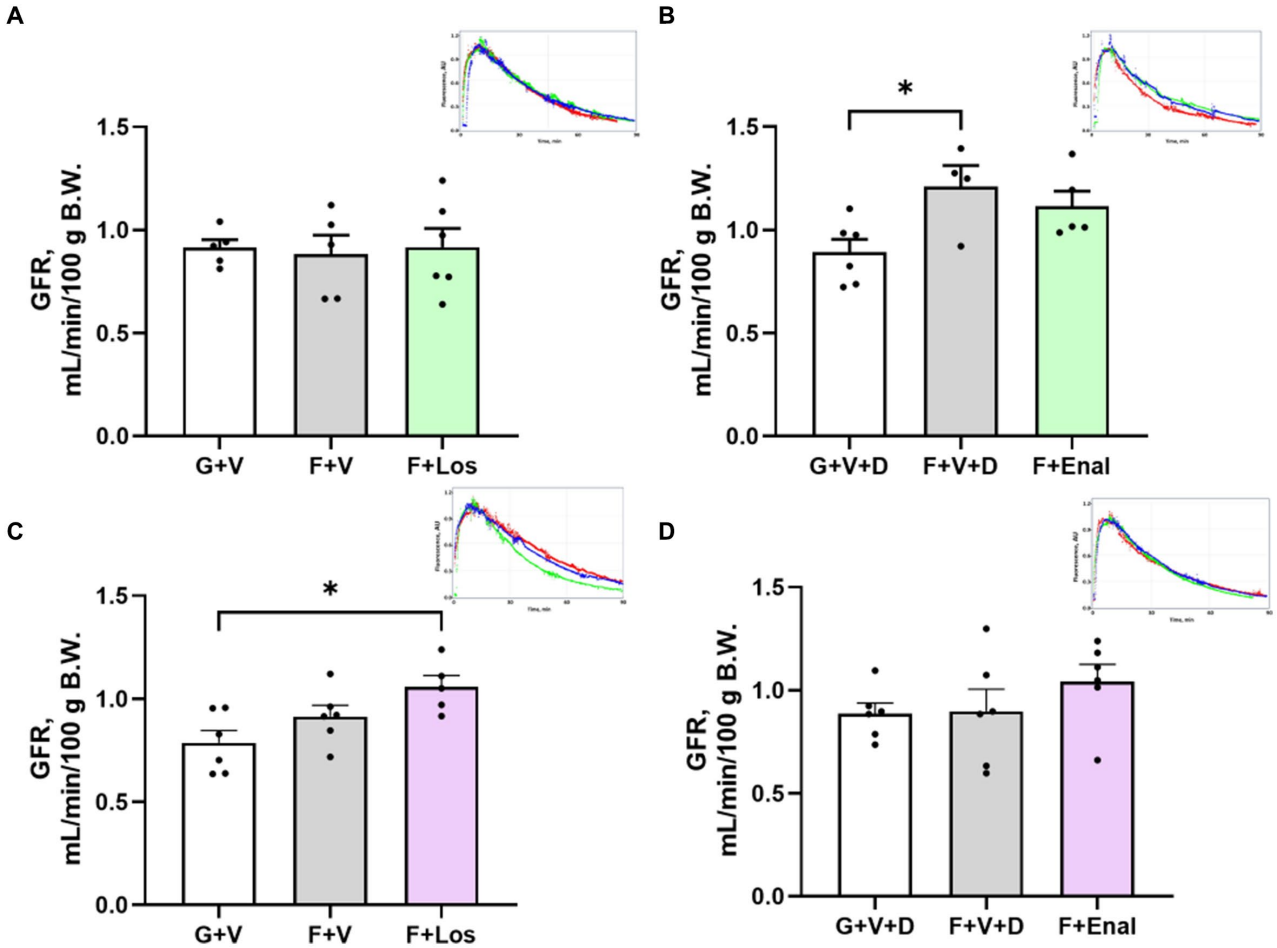
Glomerular filtration rate (GFR) in male **(A,B)** and female **(C,D)** rats fed for 3 weeks on 20% glucose plus 0.4% NaCl (G) or 20% fructose plus 4% NaCl chow (F) and concurrently treated with either saline vehicle (V) or losartan (Los, 8 mg/kg/day s.c.): G + V, F + V, and F + Los; or saline:DMSO vehicle (V + D) or enalapril (Enal, 4 mg/kg/day s.c.): G + V + D, F + V + D, and F + Enal by osmotic minipump. Insets are examples of single GFR determinations for glucose (blue), fructose (red) and drug treatment (green) in each set. Values are mean ± SE; *n* = 5–6 for each group. ^*^p < 0.05 by ANOVA.

GFR, assessed as the kinetics of the disappearance of FITC-sinistrin (Figures 5A–D), was significantly higher in male vehicle-treated fructose plus high salt rats compared with glucose plus normal salt rats (Figure 5B). Female rats in the F + Los group displayed significantly greater GFR than G + V rats (Figure 5C). Enalapril treatment in females did not achieve significance due to one rat with low GFR (Figure 5D).

**Figure 6:**
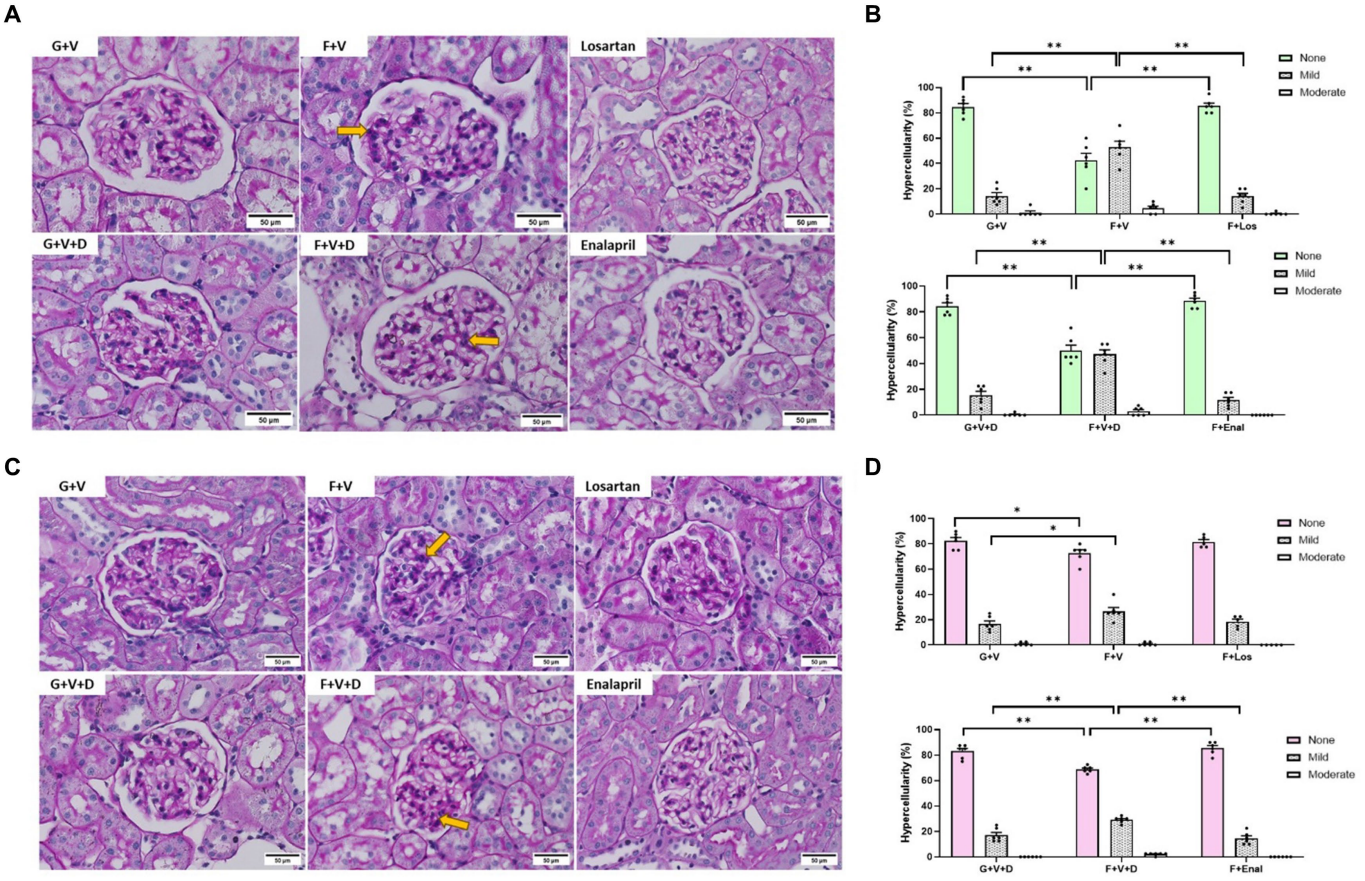
Periodic acid Schiff staining of representative glomeruli in male **(A)** and female **(C)** rats for 3 weeks on 20% glucose plus 0.4% NaCl (G) or 20% fructose plus 4% NaCl chow (F) and concurrently treated with either saline vehicle (V) or losartan (Los, 8 mg/kg/day s.c.): G + V, F + V, and F + Los; or saline:DMSO vehicle (V + D) or enalapril (Enal, 4 mg/kg/day s.c.): G + V + D, F + V + D, and F + Enal by osmotic minipump. Quantitative morphometry of mesangial hypercellularity is depicted for males **(B)** and females **(D)**. Yellow arrows indicate areas of increased mesangial hypercellularity. None, < 4; mild 4–5; moderate, 6–7; severe ≥8 mesangial cells/mesangial area. No severe glomeruli were observed. Values are mean ± SE, *n* = 6. ^*^p < 0.05 and ^**^p < 0.01 by ANOVA.

Representative renal histology for male and female rats is shown in Figures 6A,C, respectively. Quantitative mesangial morphometry is depicted in Figures 6B,D. Fructose plus high salt diet resulted in a greater proportion of glomeruli exhibiting mesangial proliferation in both males and females but was more evident in male rats (Figures 6B,C). In male rats, both losartan and enalapril decreased mesangial hypercellularity similar to that in control glucose-fed rats (Figure 6B). In contrast, enalapril but not losartan decreased mesangial hypercellularity in female fructose plus high salt-fed rats (Figure 6D).

### 3.5 Urine albumin and creatinine ratio

**Figure 7:**
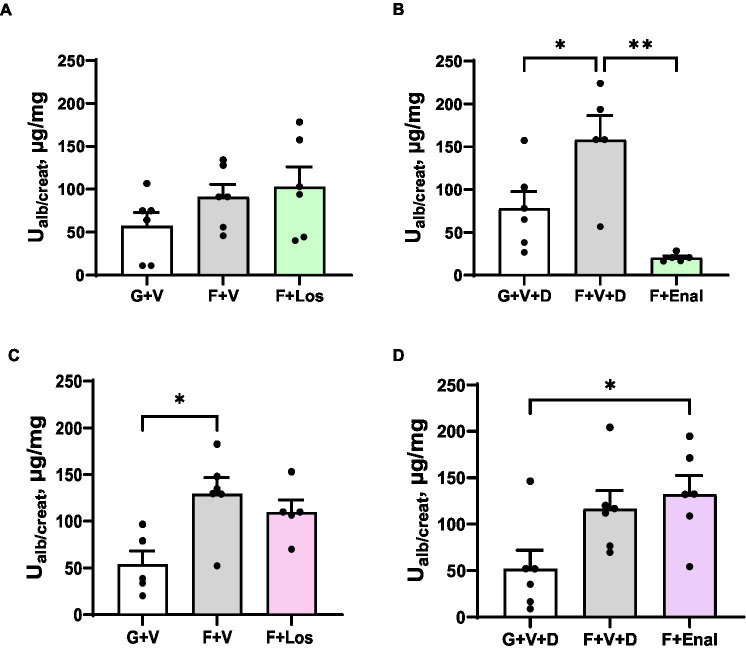
Urinary albumin to creatinine ratio (Ualb/create) in in male **(A,B)** and female **(C,D)** rats fed for 3 weeks on 20% glucose plus 0.4% NaCl (G) or 20% fructose plus 4% NaCl chow (F) and concurrently treated with either saline vehicle (V) or losartan (Los, 8 mg/kg/day s.c.): G + V, F + V, and F + Los; or saline:DMSO vehicle (V + D) or enalapril (Enal, 4 mg/kg/day s.c.): G + V + D, F + V + D, and F + Enal by osmotic minipump. Values are mean ± SE; *n* = 6 for each group. ^*^p < 0.05 and ^**^p < 0.01 by ANOVA.

Albuminuria tended to be higher in the fructose plus high salt-fed groups but achieved statistical significance only in the male F + V + D and female F + V groups compared with their respective glucose-fed counterparts. Urine albumin:creatinine ratio was higher in female F + V + D vs. G + V + D but did not achieve significance (*p* < 0.075). If both control groups and F + V and F + V + D groups are combined, rats of both sexes display significantly higher albumin:creatinine ratios with fructose plus high salt diet: 67.7 ± 12.3 vs. 121.6 ± 17.8 μg/mg [males (G + V and G + V + D) vs. (F + V and F + V + D), *p* < 0.02]; 52.7 ± 12.3 vs. 122.9 ± 12.7 μg/mg [females (G + V and G + V + D) vs. (F + V and F + V + D), *p* < 0.01]. Enalapril significantly diminished proteinuria in male rats fed a high fructose plus high salt diet (Figure 7).

## 4 Discussion

In this study, we hypothesized that salt-sensitive blood pressure and cardiorenal parameters induced by high fructose and high salt diet in Sprague Dawley rats would improve with RAS inhibition designed to restore arterial pressure to levels similar to control rats fed glucose and normal salt diet. The elevated mean arterial pressures in both male and female rats fed fructose and high salt are largely due to increases in systolic blood pressure except in fructose-fed male rats given the vehicle that contained DMSO where both systolic and diastolic pressures were elevated. The major findings of this study are that AT_1_R antagonism but not ACE inhibition decreases aortic stiffness, whereas ACE inhibition augmented cardiac output in female fructose high salt-fed rats. Nonetheless, both enalapril and losartan substantially diminish cardiac fibrosis in both sexes. Higher GFRs occur only in male fructose plus high salt-fed rats receiving the saline-DMSO vehicle, the same group that displayed elevations in both systolic and diastolic blood pressures and elevated glucose:insulin ratio. This is consistent with extensive data showing renal hyperfiltration in pre-and early diabetic rats and humans (44). The mechanism underlying higher GFR in the fructose-high salt female rats with Ang II inhibition is less clear, but likely due to improved glomerular dynamics. Notably, mesangial hypercellularity is evident in both sexes on the fructose high salt diet but is most prominent in male rats regardless of the vehicle infused. Albuminuria tends to parallel the findings in mesangial hypercellularity with a profound decrease in male rats treated with enalapril.

### 4.1 Fructose and high salt: blood pressure and insulin resistance

Our findings are in alignment with previous studies showing that intake of a diet enriched in fructose and high salt results in higher blood pressures, an effect consistently observed in male rats (19, 20, 28, 45–47). The impact of such a diet on female rats is less consistent with some studies showing no change in blood pressure (30) and others demonstrating an increase in systolic blood pressure (48). Even within the present study, systolic and mean blood pressures are higher in one group of female rats fed fructose plus high salt diet and not in the other group. A role for varying effects of vehicle treatments cannot be discounted and provides a rationale for not combining the different vehicle-treated groups of either sex.

Additional factors may influence the impact of fructose on blood pressure. The approach used is important. Blood pressures assessed by plethysmography are higher (20, 48–50) compared with blood pressures obtained by direct arterial catheter or telemetry in freely moving rats within their home cages (19, 30, 45, 48). The influence of plethysmography on male rats appears to be greater compared with female rats (48). The present study was not designed to assess the role between sexes in the blood pressure response to fructose plus high salt diet; nevertheless, if such studies are undertaken consideration should be given to the potential confounding influences of methodology in evaluating differences in blood pressure between sexes.

The mode of delivery of dietary fructose may also be a factor in both blood pressure and insulin sensitivity. When fructose is incorporated into the chow, the proportion of calories provided by fructose can be rigorously controlled, thereby more closely mimicking the percentage of fructose calories ingested in the highest quintile of western population (51). When 20% (w/v) fructose is provided in the drinking water along with high salt diet in rats, the proportion of energy intake as fructose can vary from 40 to 58% of total daily calories (20). Since sodium absorption by the gut and reabsorption by renal tubular transporters is stimulated by fructose (52, 53), it is conceivable that the greater quantity of dietary fructose provided in drinking water when combined with high salt diet may exert a greater influence on blood pressure and other cardiorenal factors than when the fructose is present in chow.

Insulin resistance with fructose ingestion in rodents has been reported by other investigators (54) especially when fructose is combined with high salt diet (55) or for extended periods of time (56). In contrast, a difference in insulin sensitivity as measured by glucose:insulin ratio is evident only in the fructose-high salt male rats with vehicle plus DMSO compared with studies from our laboratory when fructose is provided in the drinking water (28, 29) or for longer periods of time in chow (47).

These factors may also apply to human consumption of fructose. Much of fructose in human diets is ingested as sugar-sweetened beverages but high fructose corn syrup is also present in a substantial number of other food products. There is evidence to suggest that dietary sugars ingested as beverages are disproportionately linked to higher blood pressure and metabolic syndrome compared with other sources fructose (57, 58).

### 4.2 Fructose and high salt: cardiovascular parameters

Several studies have shown that a fructose-rich diet blunts the suppression of renin secretion by high salt diet and results in higher plasma Ang II (20, 29, 46, 59). Although increased plasma concentrations of RAS components have not been universally observed (34), augmented expression of ACE and AT_1_R in cardiac and aortic tissue has been observed (36, 60). Enhanced cardiac collagen deposition occurs in male rodents with prolonged fructose feeding (61, 62) or higher dietary fructose content (31). Cardiac fibrosis has also been reported in female rats fed high fructose diet (63). More extended periods of fructose plus high salt feeding result in diastolic dysfunction (47). The present histopathologic data demonstrate that cardiac fibrosis increases even after short term fructose plus high salt diet and prior to the ability to discern functional changes in left ventricular systolic or diastolic function in either male or female rats. Elevated blood pressure disrupts the equilibrium between collage types I and III synthesis and degradation resulting in collagen accumulation and myocardial fibrosis develop (64–66). Importantly, treatment with either an ACE inhibitor or AT_1_R antagonist such that blood pressure is restored to a level comparable to that in their respective glucose plus normal salt counterparts successfully mitigates cardiac collagen deposition.

Dietary fructose-induced increases in aortic collagen have been reported as early as 1968 (67). PWV as an index of vascular stiffness is highly predictive of cardiovascular morbidity and mortality in humans (68, 69). Notably, aortic compliance in male but not female rats is diminished when given 20% fructose in their drinking water for 3 weeks (29, 30). A role for acute inhibition of sympathetic excitation (29) or enhancement of vagal activity (70) to improve vascular function with fructose-associated cardiometabolic alterations has been identified. Despite greater ACE and AT_1_R mRNA expression (60) and protein content (36) in aortic tissue, acute inhibition of RAS fails to alter PWV, an index of aortic stiffness in fructose plus high salt fed rats (30). These results contrast with the improved vascular remodeling and stiffness with RAS inhibition in other models such as the spontaneously hypertensive rat (71), streptozotocin-induced diabetes (72), cystic renal disease (73) and iron-overload (74). Notably, aortic stiffness is not augmented within the 3-week timeframe in either sex with dietary fructose provided in rat chow rather than drinking water, nor is PWV altered with chronic RAS inhibition. It remains to be seen whether PWV may benefit from ACE or AT_1_R blockade in rats fed this diet for longer periods of time when aortic stiffness is enhanced (47). Alternatively, other aberrations in factors such as NADPH oxidase, nitric oxide, and oxidative stress may contribute to disordered vascular compliance in this model independent of the RAS pathway (75).

### 4.3 Fructose and high salt: renal parameters

Albuminuria is an early index of renal injury. Elevated albumin excretion has been observed in male fructose-fed Dahl salt-sensitive rats (34) as well as Sprague Dawley rats fed fructose and high salt for 12 weeks (19). Albumin excretion shows greater variability in the current experiments since the study was not powered to evaluate albuminuria. When the groups are combined regardless of vehicle treatment, albuminuria is clearly augmented in the fructose plus high salt-fed groups of each sex. Moreover, RAS inhibition exerts a greater beneficial effect on albuminuria in male rats. The impact on albuminuria by fructose as well as RAS inhibition is consistent with the observed changes in mesangial hypercellularity which was more evident in the male rats. Mesangial cell proliferation occurs in response to activation of hexosamine flux which typically occurs under conditions of hyperglycemia (76, 77). Fructose may also enter the hexosamine pathway by action of glutamine: fructose-6-phosphate amidotransferase (GFAT) and may be augmented on a high fructose diet. Notably, Ang II activates the GFAT promoter in mesangial cells (78) and leads to oxidative stress (79). Importantly, the mesangial hypercellularity and albuminuria are evident at a time when GFR is not yet reduced. These findings are consistent with data showing glomerular hypertension and elevated single nephron GFR with a high fructose diet (80). Indeed, more prolonged ingestion of a fructose plus high salt diet lowers GFR (19). Due to the association of fructose and high salt diet with elevated blood pressure, most studies on fructose effects in the kidney have focused on tubular sodium transport (53), but further investigations of a role in glomerular function and histopathology are also warranted.

### 4.4 Limitations

As noted above, there is a considerable difference in fructose calories when the fructose is delivered in the drinking water vs. rat chow such that the percentage of fructose calories provided with rat chow is specifically 20% of total caloric intake. If the data from Gordish et al. (20) is generalizable, when fructose is provided as 20% w/v in the drinking water the proportion of fructose calories is much higher. This may account for the lack of significant effects on insulin resistance, PWV, diastolic LV function and renal function observed in the fructose high salt-fed groups compared with our previous study where fructose was in the drinking water (30). A longer-term study of fructose and high salt exposure would be ideal; however, the mode, timing, and duration of delivery of RAS inhibitors will need to be carefully considered. The use of osmotic minipump delivery is superior giving the drugs in the drinking water especially as water intake may vary with fructose and salt intake making dosing irregular. On the other hand, a long-term study such as 12-weeks would require repeated minipump replacements. In addition, blood for evaluation of glucose and insulin was obtained from conscious animals to avoid changes imposed by the type or depth of anesthesia. This also limited the volume of blood that could be acquired without causing hypotension and influencing results and, in some cases, the catheters were occluded so that samples could not be obtained. A more rigorous measure of insulin sensitivity would have been the hyperinsulinemic euglycemic glucose clamp test; however, insulin is known to stimulate Ang II and to alter the actions of angiotensin peptides (81). Thus, the insulin infusion could have potentially altered parameters in our primary hypothesis, namely the RAS pathway so we chose the glucose:insulin ratio as an estimate of insulin sensitivity. Since urine for albumin and creatinine was obtained directly from the bladder at the time of harvesting, some samples were of insufficient volume to permit assay. Ideally, it would have been good to have had measure of renal blood flow or plasma flow but approaches in conscious rats similar to that used here for GFR are not yet available. It was necessary to use two different vehicles for enalapril and losartan, respectively. The study was powered to detect changes in PWV. Due to concerns regarding possible vehicle effects especially with the DMSO, this precluded combining the groups not treated with RAS inhibition and thus diminished power for some of the other measurements.

## 5 Conclusion

In summary, these data confirm earlier findings that as little as 3 weeks of high fructose and high salt diet results in elevated blood pressure in both male and female rats. This short-term diet regimen does not change PWV, LV function or GFR in either sex. RAS blockade with either an ACE inhibitor or AT_1_R antagonist is able to restore MAP to levels fructose high salt-fed rats similar to control glucose-fed rats. The only improvement in PWV occurs in female rats treated with losartan. Despite the lack of change in functional cardiorenal parameters, significant histopathologic changes occur in both cardiac and renal mesangial tissues and are ameliorated by RAS inhibition. Urinary albumin excretion is a marker of glomerular dysfunction prior to the development of frank decline in renal function and is also ameliorated by RAS inhibition in male rats.

## Data Availability

The raw data supporting the conclusions of this article will be made available by the authors, without undue reservation.
