# Supplementary material for: Impact of inhibition of the renin-angiotensin system on early cardiac and renal abnormalities in Sprague Dawley rats fed short-term high fructose plus high salt diet
Source: Front Nutr. 2024 Aug 22;11:1436958. doi: 10.3389/fnut.2024.1436958 (PMC11376227; doi:10.3389/fnut.2024.1436958)
Supplement: Supplementary file 2 [file Table_2.DOCX]

**Table S2**. Test Diet 20% Fructose, 1.1% K, 4.0% Na, Red

| **Protein, %** | 18.6 | **Minerals** |  |
| --- | --- | --- | --- |
| Arginine,% | 0.73 | Calcium,% | 0.6 |
| Histidine,% | 0.54 | Phosphorus, % | 0.56 |
| Isoleucine,% | 1 | Potassium, % | 1.22 |
| Leucine,% | 1.82 | Magnesium, % | 0.07 |
| Lysine,% | 1.53 | Sodium, % | 4 |
| Methionine,% | 0.69 | Chloride, % | 6.85 |
| Cystine, % | 0.08 | Fluorine, ppm | 5 |
| Phenylalanine, % | 1 | Iron, ppm | 68 |
| Tyrosine,% | 1.06 | Zinc, ppm | 27 |
| Threonine, % | 0.81 | Manganese, ppm | 65 |
| Tryptophan, % | 0.23 | Copper, ppm | 15 |
| Valine, % | 1.2 | Cobalt, ppm | 3.2 |
| Alanine,% | 0.58 | Iodine, ppm | 0.57 |
| Aspartic Acid, % | 1.35 | Chromium, ppm | 3 |
| Glutamic Acid, % | 4.29 | Molybdenum, ppm | 0.82 |
| Glycine, % | 0.41 | Selenium, ppm | 0.3 |
| Proline, % | 2047 |  |  |
| Serine, % | 1.16 | **Vitamins** |  |
| Taurine, % | 0 | Vitamin A, IU/g | 22.1 |
|  |  | Vitamin D-3, IU/g | 2.2 |
| **Fat, %** | 10 | Vitamin E, IU/kg | 55.2 |
| Cholesterol, ppm | 48 | Vitamin K, ppm | 10.4 |
| Linoleic Acid, % | 3.34 | Thiamin, ppm | 18.8 |
| Linolenic Acid, % | 0.07 | Riboflavin, ppm | 20.7 |
| Archidonic Acid, % | 0.01 | Niacin, ppm | 90 |
| Omega-3 Fatty Acids, % | 0.07 | Pantothenic Acid, ppm | 56 |
| Total Saturated Fatty Acid, % | 2.72 | Folic Acid, ppm | 4.2 |
| Total Monosaturated Fatty Acid, % | 3.31 | Pyridoxine, ppm | 16.5 |
| Polyunsaturated fatty Acid, % | 3.42 | Biotin, ppm | 0.4 |
| **Fiber(max), %** | 4.6 | Vitamin B-12, mcg/kg | 23 |
| **Carbohydrates, %** | 49.1 | Choline Chloride, ppm | 1400 |
| **Energy (kcal/g)** | 3.61 | Ascorbic Acid, ppm | 0 |
