# Supplementary figures and images for: Impact of inhibition of the renin-angiotensin system on early cardiac and renal abnormalities in Sprague Dawley rats fed short-term high fructose plus high salt diet
Source: Front Nutr. 2024 Aug 22;11:1436958. doi: 10.3389/fnut.2024.1436958 (PMC11376227; doi:10.3389/fnut.2024.1436958)

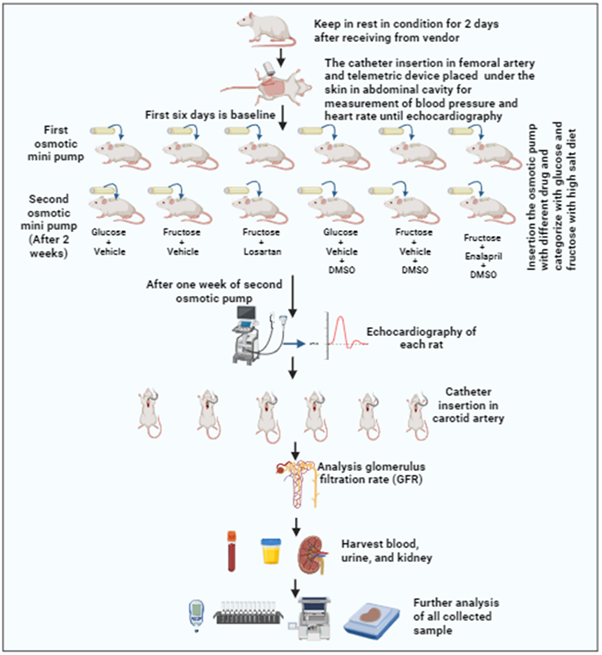

Supplement: Supplementary file 3 [file Image_1.PNG]
